# Novel evidence for a PIWI-interacting RNA (piRNA) as an oncogenic mediator of disease progression, and a potential prognostic biomarker in colorectal cancer

**DOI:** 10.1186/s12943-018-0767-3

**Published:** 2018-01-30

**Authors:** Wenhao Weng, Na Liu, Yuji Toiyama, Masato Kusunoki, Takeshi Nagasaka, Toshiyoshi Fujiwara, Qing Wei, Huanlong Qin, Haifan Lin, Yanlei Ma, Ajay Goel

**Affiliations:** 1 Center for Gastrointestinal Research; Center for Translational Genomics and Oncology, Baylor Scott & White Research Institute and Charles A Sammons Cancer Center, Baylor University Medical Center, 3410 Worth Street, Suite 610, Dallas, TX 75246 USA; 2 Department of Clinical Laboratory, Yangpu Hospital, Tongji University School of Medicine, Shanghai, China; 3 Center for Translational Medicine, Yangpu Hospital, Tongji University School of Medicine, Shanghai, China; 4 Department of Cell Biology; Department of Genetics; Department of Obstetrics, Gynecology, and Reproductive Sciences; Yale Stem Cell Center, Yale University School of Medicine, New Haven, CT USA; 5 Department of Gastrointestinal and Pediatric Surgery, Division of Reparative Medicine, Institute of Life Sciences, Mie University Graduate School of Medicine, Mie, Japan; 6 Department of Gastroenterological Surgery and Surgical Oncology, Okayama University Graduate School of Medicine, Dentistry and Pharmaceutical Sciences, Okayama, Japan; 7 Department of Pathology, Shanghai Tenth People’s Hospital, Tongji University, Shanghai, China; 8 Department of GI Surgery, Shanghai Tenth People’s Hospital Affiliated to Tongji University, Shanghai, China; 9 Department of Colorectal Surgery, Fudan University Shanghai Cancer Center, 270 Dong’an Road, Shanghai, 20032 China; 10 Department of Oncology, Shanghai Medical College, Fudan University, 270 Dong’an Road, Shanghai, 20032 China

**Keywords:** Colorectal cancer, piRNA, piR-1245, Prognosis, Biomarker, Noncoding RNA, Tumor suppressor, Oncogene

## Abstract

**Background:**

Emerging evidence suggests that PIWI-interacting RNAs (piRNAs) may be important epigenetic regulators of gene expression in human cancers; however, their functional and clinical significance in colorectal cancer (CRC) remains unknown.

**Methods:**

We performed piRNA expression profiling in paired cancer and normal tissues through small RNA-sequencing. The clinical significance of candidate piRNAs was investigated, and independently validated in 771 CRC patients from three independent cohorts. The biological function of piRNAs was characterized in cell lines, followed by identification and validation of downstream target genes in CRC tissues.

**Results:**

We identified piR-1245 as a novel and frequently overexpressed noncoding RNA in CRC, and its expression significantly correlated with advanced and metastatic disease. Patients with high piR-1245 expression experienced significantly shorter overall survival, and multivariate analysis identified its expression to serve as an independent prognostic biomarker in CRC. Functionally, piR-1245 acts as an oncogene and promotes tumor progression, and gene expression profiling results identified a panel of downstream target-genes involved in regulating cell survival pathway. Based upon piRNA:mRNA sequence complementarity, we identified a panel of tumor suppressor genes (ATF3, BTG1, DUSP1, FAS,NFKBIA, UPP1, SESN2, TP53INP1 and MDX1) as direct targets of piR-1245, and successfully validated an inverse correlation between their expression and piR-1245 in CRC.

**Conclusions:**

We for the first time have identified the role for a PIWI-interacting noncoding RNA, piR-1245, as a novel oncogene and a potential prognostic biomarker in colorectal cancer.

**Electronic supplementary material:**

The online version of this article (10.1186/s12943-018-0767-3) contains supplementary material, which is available to authorized users.

## Background

Colorectal cancer (CRC) constitutes a major public health burden [1], being the third most commonly diagnosed cancer, and the fourth leading cause of cancer-related deaths worldwide. Interestingly, recent reports have shown that the incidence of colorectal cancer in Asian countries, which historically was relatively low, has increased dramatically during the last two decades [2, 3]. Considering the high disease burden and mortality associated with this global disease, it is imperative to develop effective prevention and treatment strategies for the management of patients suffering from this malignancy.

CRC develops as a consequence of stepwise accumulation of multiple genetic and epigenetic alterations, which occur with tumor initiation and ensue during disease progression. In view of tumor heterogeneity, the prognosis and response to chemotherapy between individual patients can vary significantly. However, current guidelines for risk stratification of patients predominantly rely on the clinicopathological factors, which are inadequate and often result in under or over-treatment for CRC patients [4, 5]. In view of this clinical challenge, identification of novel molecular targets that more robustly typify and represent disease biology would be of great value in improving prognosis and allowing precision therapeutic targeting in CRC patients.

Data gathered during the last decade has revealed that microRNAs play a crucial role in cancer pathogenesis, and may serve as promising disease biomarkers and potential therapeutic targets [6, 7]. Meanwhile, emerging data from RNA-Sequencing efforts of cancer specimens have led to the discovery of additional classes of novel, small noncoding RNAs (ncRNAs), which may also significantly contribute to cancer pathogenesis [8–12]; however, such data remain in their infancy at this time in point. Among these, PIWI-interacting RNAs (piRNAs), represent the most diversified group, but currently remain the least characterized class of small ncRNAs. The piRNA pathway consists of piRNAs that interact with PIWI proteins, in which the precursor piRNAs are transcribed from their clusters, cleaved by PIWI proteins, and subsequently amplified in the cytoplasm through sequence-complementary-dependent cycle. The piRNA-PIWI protein pathway was initially found associated with safeguarding of the germline genome against transposon-induced insertional mutations [13–15]. However, emerging evidence indicates that piRNAs may also function on a somatic level, whereby they regulate gene expression; through histone modifications and DNA methylation [16–19]. In other words, although such recent evidence suggests the role of PIWI-piRNA pathway in controlling epigenetic function, our understanding for the biological involvement of piRNAs in human cancers currently remains in its infancy, but presents an exciting new area of basic and translational research worthy of exploration.

Although piRNA-mediated gene expression regulation may have a broader implication for cancer research, recent studies have largely been limited to expression profiling of a handful or selected, small subset of piRNAs in different cancer types. [9, 12] Furthermore, the role of piRNAs in CRC is poorly understood. Hence, we envisaged this first of its kind of study to systematically and comprehensively interrogate the molecular contributions of piRNAs in CRC, with a goal to identify novel, differentially expressed piRNAs that promote colorectal carcinogenesis, and decipher whether these piRNAs may have translational relevance as clinically relevant disease biomarkers. Accordingly, we performed a discovery step by performing small RNA-Sequencing-based expression profiling for piRNAs between cancer and normal tissues. Using a series of bioinformatic approaches, we identified candidate, CRC-specific piRNAs, followed by their validation in multiple CRC patient cohorts. We subsequently supported these findings by performing a series of functional assays and investigated downstream pathways and target genes of candidate piRNAs, which contribute to the neoplastic progression in colorectal cancer.

## Methods

### Patients and study design

To identify CRC-associated piRNAs, we performed small RNA-sequencing on a subset of frozen cancer tissues and paired normal mucosa (NM) specimens (4 each), which were collected at the Mie University, Japan. To confirm the expression levels of candidate piRNAs between cancer and normal tissues, we measured their expression in matched cancer and normal tissues in three independent patient cohorts from the Mie University, Japan (*n* = 20), Shanghai Tenth People’s Hospital, China (*n* = 20) and Okayama University Medical Hospital, Japan (*n* = 18). To investigate the prognostic potential of candidate piRNAs in CRC, we analyzed piR-1245 expression pattern in three different patient cohorts with a combined total of 771 CRC patients from the TCGA dataset, a clinical testing cohort and an independent validation cohort. The expression profiles of piRNAs from the TCGA (The Cancer Genome Atlas) dataset (*n* = 387) was characterized by Martinez, et al. [20]. We thereafter analyzed expression of candidate piRNAs in a clinical testing cohort (*n* = 195, Shanghai Tenth People’s Hospital) and an independent validation cohort (*n* = 189, Okayama University Medical Hospital). Both testing and validation cohort were formalin fixed paraffin embedded tissue specimens, which allowed us to perform micro-dissection for enriching the RNA content from the neoplastic cells. The baseline characteristic of these patient cohorts is described in Table 1. To further understand the mechanistic correlation of piRNAs with its downstream target genes, we evaluated their expression in a cohort of 159, fresh frozen tissues. Written informed consent was obtained from all patients, and the study was approved by the institutional review boards of all participating institutions. All CRC patients were followed up for survival for at least 5 years from their date of surgery. Patients treated with radiotherapy or chemotherapy before surgery were excluded from the study.

**Table 1:** Clinicopathological characteristic and piR-1245 expression in training and validation cohort

	Training cohort				Validation cohort			
	Cases	Low	High	*P* ^*c*^	Cases	Low	High	*P* ^*c*^
Gender								
Male	91	45	46	0.9391	110	52	58	0.4255
Female	104	52	52		79	42	37	
Age								
≤ 69^a^/66^b^	100	55	45	0.133	100	45	55	0.1687
> 69^a^/66^b^	95	42	53		89	49	40	
Tumor location								
Distal	150	82	68	*0.0123	121	55	66	0.1174
Proximal	45	15	30		68	39	29	
Histological type								
Well/moderate	175	90	85	0.0566	180	90	90	0.7456
Poor	18	5	13		9	4	5	
Unknown	2	–	–		–	–	–	
Pathological T category								
pT1-3	48	34	14	**0.0008	154	82	72	*0.0434
pT4	147	63	84		35	12	23	
Lymph node metastasis								
Negative	132	73	59	*0.025	85	53	32	**0.0025
Positive	63	24	39		100	40	60	
Unknown	–	–	–		4	–	–	
Distant metastasis								
Negative	187	96	91	*0.0319	143	80	63	**0.0027
Positive	8	1	7		46	14	32	
Stage								
I	29	21	8	**0.006	28	18	10	**0.0052
II	99	51	48		53	33	20	
III	59	24	35		62	29	33	
IV	8	1	7		46	14	32	

Boldface *P* value denotes statistical significance  < 0.05

^a^The median age of training cohort is 69

^b^The median age of validation cohort is 66

^c^Pearson chi-squared testing was used - compare the correlation between piR-1245 expression and clinical variables. *P < 0.05;**P < 0.01

### Small RNA-sequencing, piRNA quantification and gene expression analysis

For RNA-sequencing, 1 μg of total RNA was used for library preparation with Illumina’s TruSeq small RNA sample preparation Kit using manufacturer’s recommended protocols and the previously published articles mirBase [21, 22]. Expression of identified piRNAs was analyzed using Custom TaqMan small RNA assays as described previously [23–25]. The average expression levels of tissue piRNAs was normalized against U6 using the 2^-ΔCt^ method. The relative expression of target genes was determined by 2^-Δct^ method using GAPDH as a normalizer as described in details in the Additional file 1: Supplementary Methods and primer sequences shown in Additional file 2: Table S1.

### Cell lines, RNA oligos, antisense and transfection

HCT116 and SW480 were obtained from the American Type Culture Collection (ATCC, Rockville, MD) and cultured in Iscove’s modified Dulbecco’s medium (Invitrogen, Carlsbad, CA). For the overexpression of piR-1245, both cells lines were transfected in triplicates with either single-stranded RNA oligos or scrambled RNA controls. For the inhibition of piR-1245 in CRC cell lines, we designed antisense oligos as described previously [26] and as described in the Additional file 1: Supplementary Methods.

### MTT, colony formation, cell invasion, migration and apoptosis assays

MTT, colony formation, invasion, migration and apoptosis assays were performed in CRC cell lines at different time points using standard approaches, and according to the manufacturer’s instructions, as described in the Additional file 1: Supplementary Methods.

### Immunofluorescence (IF) staining

For IF, cells were fixed by 4% paraformaldehyde for 15 min, washed with PBS and blocking buffer (3% FBS, 1% heat-inactivated sheep serum, 0.1% Triton X-100), and thereafter incubated overnight at 4 °C with primary antibodies against Ki-67 (Santa Cruz, Dallas, TX), and fluorescent Alexa Fluor 488- conjugated secondary antibodies (Thermo Scientific, Rockford, IL) were subsequently used for fluorescence detection. The Ki-67 staining intensity was semi-quantified as follows: – for negative staining, ± for very weak staining, + for weak staining and ++ for strong staining.

### Gene expression microarray analysis

To investigate the regulatory role of piR-1245 on genome-wide target mRNAs, we treated HCT116 cells with or without piR-1245 antisense, and subsequently performed Affymetrix GeneChip Human gene 2.0 ST arrays and subsequent bioinformatic analysis as described in the Additional file 1: Supplementary Methods.

### piRNA target prediction

Based upon piRNA:mRNA sequence complementarity, we used Miranda v3.3a and RNA22 program to search for targets of piR-1245 against all human transcripts. The candidate piRNAs were selected based on prediction scores and binding energy. The whole transcript region of human transcripts were used for piRNA target prediction.

### Statistical analysis

All statistical analyses were performed using the GraphPad Prism version 6.0 or MedCalc version 12.3 programs. Statistical differences between groups were determined by Wilcoxon’s signed rank test, the χ2 test or Mann-Whitney U test. Kaplan-Meier analysis and log-rank test was used to estimate and compare overall survival (OS) rates of CRC patients with high and low piR-1245 expression. The optimal cutoff values were determined by ROC curves to discriminate patients with or without death. The Cox’s proportional hazards models were used to estimate hazard ratios (HRs) for death. All *P* values were 2-sided, and those less than 0.05 were considered statistically significant.

## Results

### Identification of cancer-related piRNAs in CRC

**Fig. 1:**
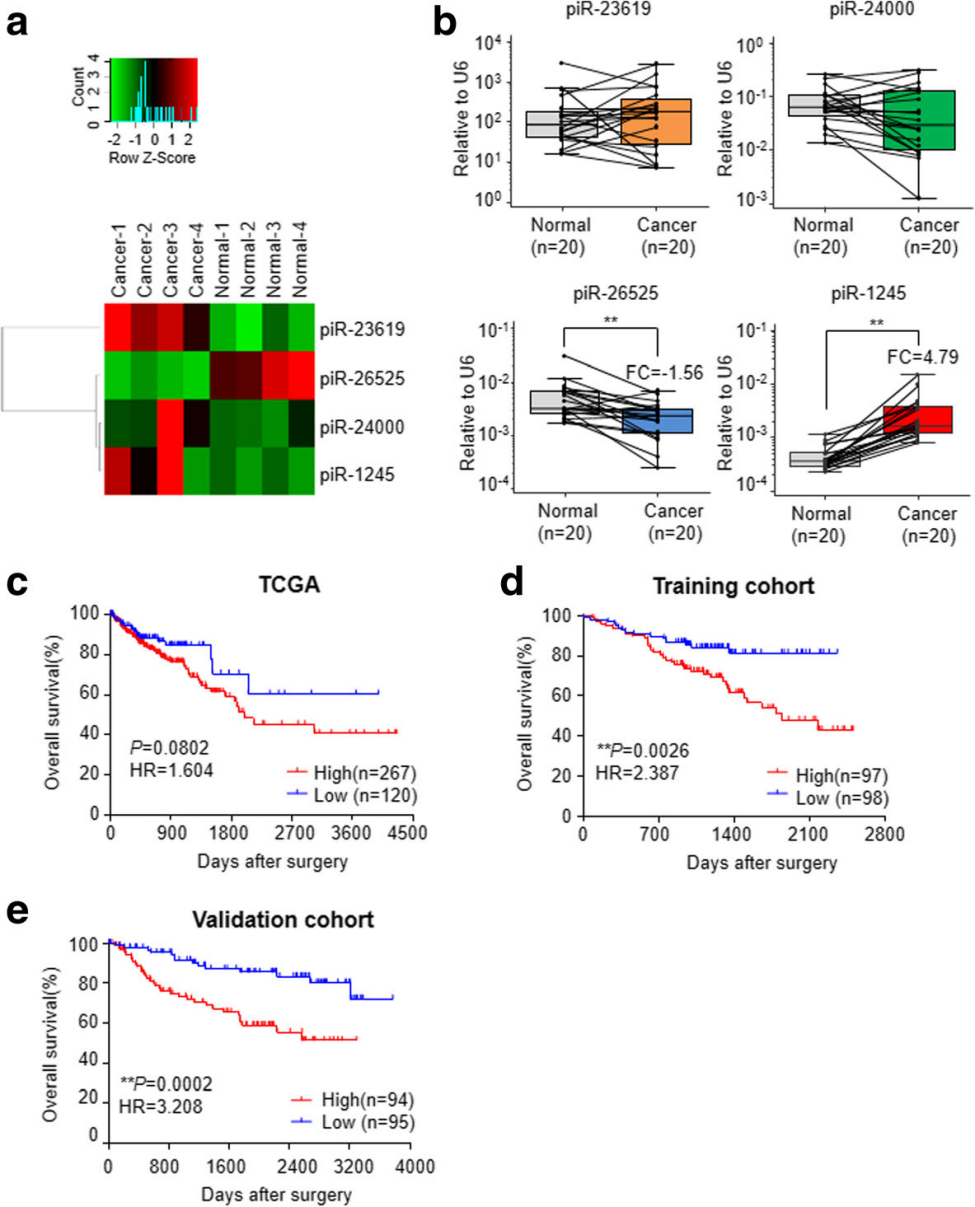
The clinical significance of cancer-related piRNAs in CRC. **a** Small RNA-seq revealed differentially expressed piRNAs between cancer and paired normal tissues (4 each) from the Mie cohort. The heatmaps maps illustrate the Z-score of each candidate piRNAs. **b** The expression of candidate piRNAs were validated in a subset of 20 cancer and paired normal mucosa (NM) specimens in Mie cohort. **P < 0.01, Wilcoxon paired test. The prognostic significance of piR-1245 was evaluated in colorectal cancer patients from TCGA datasets (**c**) and the clinical training cohort (Shanghai) (**d**) and the validation cohort (Okayama). **e** The OS (overall survival) analysis was performed by Kaplan–Meier test and the log-rank method (**P < 0.05, HR: Hazard Ratio)

PIWI proteins, central to piRNA biogenesis, have recently been found to be frequently overexpressed in different cancer types [27–31]. Interestingly, through a systematic query of the Oncomine [32] and Protein Atlas database [33–36], we noted that the mRNA and protein levels of PIWIL1 and PIWIL4, two of the major PIWI proteins, are significantly overexpressed in CRC vs. normal tissues. The implications of these findings are that piRNAs may also be consequently dysregulated and involved in CRC development- a question that has not been interrogated previously (Additional file 3: Figure S1). Accordingly, we firstly performed small RNA-seq analysis to identify CRC-associated piRNAs, by analyzing a subset of CRC and paired normal mucosa (NM) specimens in the Mie cohort. Our results revealed that 3 piRNAs (piR-23,619, piR-24,000 and piR-1245) were up-regulated, while piR-26,525 was down-regulated in cancer tissues (with ≥ 2 fold change and a *P* value ≤ 0.01; Fig. 1a). To reduce sampling error, we subsequently measured expression level of these candidates in a subset of 20 cancer and paired NM specimens from the same cohort. Unfortunately, we only found piR-1245 and piR-26,525 had significant differential expression between cancer and normal tissues (Fig. 1b). Furthermore, in order to account for patient cohort differences and tumor heterogeneity, we also validated the expression of these two piRNAs in two independent patient cohorts (Shanghai and Okayama cohorts). These validation experiments confirmed that the expression of piR-1245, but not piR-26,525, was higher in CRC tissues, that its expression levels demonstrated a 5.49-fold (*P* < 0.01) and a 7.0 fold (*P* < 0.01) increase in CRC vs. NM tissue expression in the two cohorts (Additional file 4: Figure S2). These data suggested that piRNA piR-1245 was a potential oncogenic piRNA in colorectal cancer. In particular, we noticed that piR-1245 is not only overexpressed in CRC but also exhibits a pan-cancer expression pattern. In the TCGA datasets, we found that the expression of piR-1245 was upregulated in other types of cancer including lung, breast, stomach, bladder, kidney and prostate cancer, highlighting its important key role in carcinogenesis (Additional file 5: Figure S3).

### High expression of piR-1245 correlates with advanced disease and metastatic in colorectal cancer

We next examined the expression pattern of piR-1245 in the context of its clinical significance in the training cohort (Shanghai cohort, *n* = 195). The piR-1245 was overexpressed in all CRCs, and this phenomenon occurred in a stage-dependent manner (*P* = 0.006, Table 1). The tumors in the distal colon or rectum showed a much higher expression level of piR-1245 compared to the proximal neoplasms (*P* = 0.0123). Furthermore, higher expression of piR-1245 was significantly more pronounced in cancer tissues with poor differentiation (*P* = 0.0566), advanced T stage (*P* = 0.0008), lymph node metastasis (*P* = 0.025) and distant metastasis (*P* = 0.0319).

To further validate the correlation between piR-1245 expression and clinicopathological variables, we interrogated these associations in an independent patient cohort (Okayama cohort, *n* = 189). We were able to successfully validate our findings in the training cohort, as the upregulatedpiR-1245 was also associated with advanced T-stage (*P* = 0.0434), lymph node (*P* = 0.0025) and distant metastasis (*P* = 0.0027) in this second patient cohort as well. Collectively, our analyses provided evidence that expression ofpiR-1245 is not only upregulated, but also associates with specific clinicopathological features, suggesting that piR-1245plays a crucial role in the cancer pathogenesis within the colon.

### High expression of piR-1245 associates with poor prognosis in colorectal cancer patients

To further interrogate the impact of overexpression of piR-1245 and its potential impact on prognosis in CRC patients, we analyzed its expression pattern in three different cohorts with a combined total of 771 CRC patients from the TCGA dataset [20], clinical training cohort and the validation cohort. In the TCGA dataset, piR-1245-high expression group showed a strong tendency to be associated with poor OS (*P* = 0.0802, HR = 1.604; Fig. 1c). Therefore, we examined prognostic potency of piR-1245 in a training cohort that comprised of high quality tissues with complete follow-up clinical data. As expected, piR-1245-high expression group significantly correlated with poor OS (*P* = 0.0026, HR = 2.387; Fig. 1d). To further confirm the prognostic value of piR-1245 in CRC patient survival, we investigated these associations in another validation cohort, and successfully once again confirmed that piR-1245-high expression group demonstrated shorter OS (*P* = 0.0002, HR = 3.208; Fig. 1e), highlighting its clinical significance as independent prognostic biomarker in CRC patients. Furthermore, multivariate cox’s regression analysis revealed that high piR-1245 expression was an independent predictor for poor prognosis in both clinical testing and validation cohorts (HR: 1.965, 95%CI: 1.0683 to 3.6144, *P* = 0.0298, HR: 2.9347, 95%CI: 1.4584 to 5.9057, *P* = 0.0025, respectively, Table 2). Taken together, these findings elucidate that overexpression of piR-1245 has clinical significance, and can serve as a potential prognostic biomarker in CRC patients.

**Table 2:** Univariate and multivariate analysis for predictors of overall survival in training and validation cohort

	Univariate survival analysis			Multivariate survival analysis		
	HR	95%CI	*P*	HR	95%CI	*P*
Training cohort						
Gender(Male)	0.8471	0.4948-1.4500	0.545			
Age (> 69)	1.9063	1.0970-3.3125	*0.0221			
Tumor location (Proximal)	2.3263	1.3379-4.0449	**0.0028			
Histological type (Poor)	2.1597	1.0165-4.5887	*0.0452	1.945	0.9003-4.2019	0.0905
T classification (pT4)	2.6736	1.2045-5.9346	*0.0156	2.1501	0.9431-4.9017	0.0687
Node involvement (Present)	1.6608	0.9700-2.8434	0.0644	1.3642	0.7813-2.3820	0.2748
Distant metastasis (Present)	4.8339	2.0511-11.3923	**0.0003	4.796	1.9696-11.6786	**0.0006
piR-1245 expression level (High)	2.387	1.3300-4.2838	**0.0035	1.965	1.0683-3.6144	*0.0298
Validation cohort						
Gender(Male)	1.1471	0.7592-1.7334	0.5145			
Age (> 69)	1.077	0.6065-1.9124	0.8002			
Tumor location (Proximal)	0.7355	0.3972-1.3619	0.3284			
Histological type (Poor)	3.7535	1.4754-9.5487	**0.0055	3.4914	1.2996 - 9.3799	*0.0132
T classification (pT4)	3.61	2.0014-6.5114	** < **0.0001**	2.2202	1.1551 - 4.2673	*0.0167
Node involvement (Present)	1.921	1.2479-2.9573	**0.003	1.2908	0.5790 - 2.8776	0.5326
Distant metastasis (Present)	8.1136	4.4863-14.6739	** < **0.0001**	4.7427	2.3622 - 9.5220	** < **0.0001**
piR-1245 expression level (High)	3.208	1.6989-6.0578	**0.0003	2.9347	1.4584 - 5.9057	**0.0025

Boldface *P* value denotes statistical significance  < 0.05

*HR* Hazard ratio; *P < 0.05;**P < 0.01

### The piR-1245 has multiple functional roles in promoting tumor progression in colorectal cancer cells

**Fig. 2:**
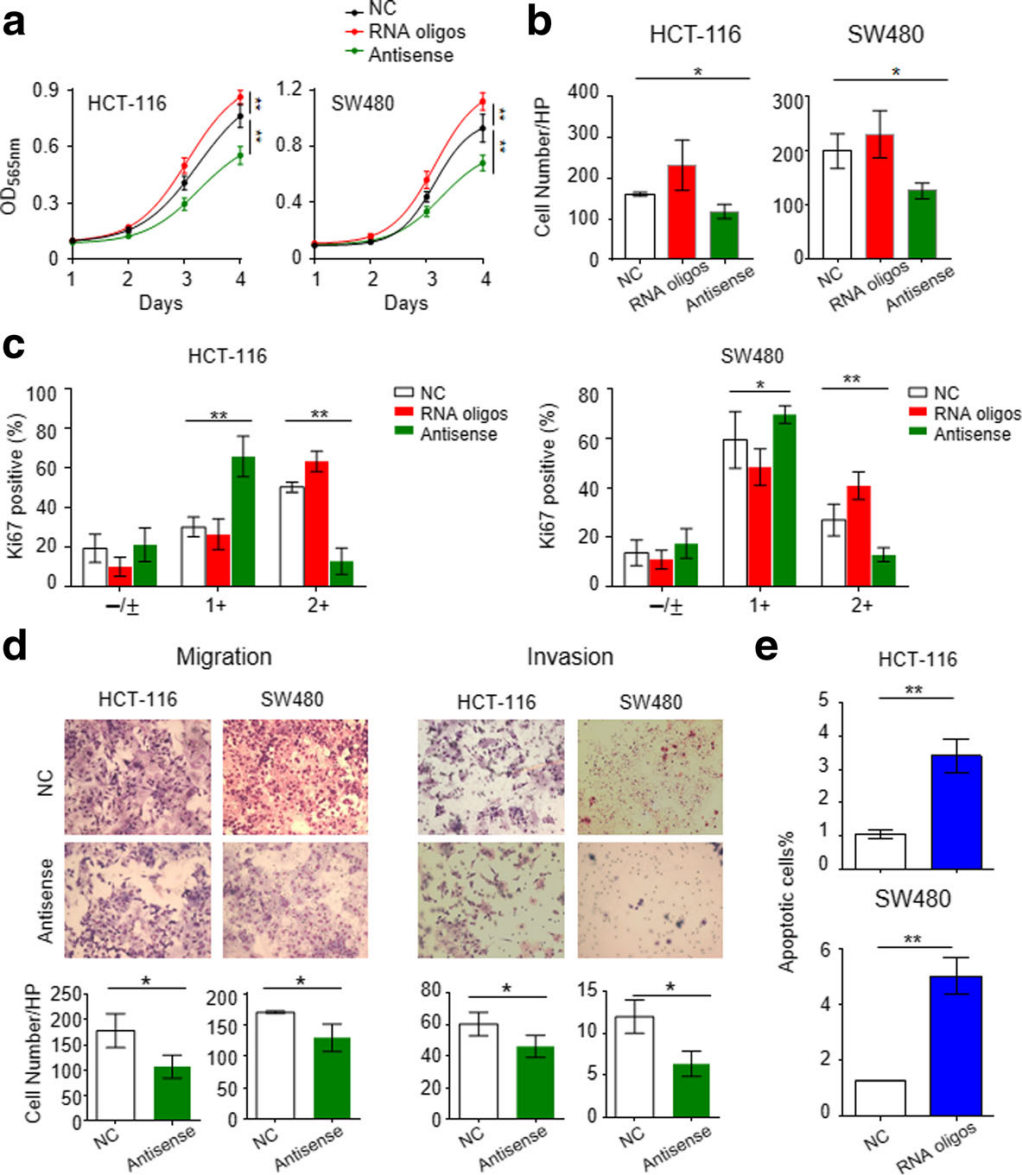
The piR-1245 promotes cell growth, colony formation, migration and invasion and inhibits apoptosis in colorectal cancer cells. HCT116 and SW480 cells were transfected with either piR-1245 RNA oligonucleotides, antisense or scrambled controls. The treated cells or control cells were subsequently used for MTT assay (**a**), colony formation assay (**b**), Ki-67 staining (**c**), Migration and invasion assay (**d**) and apoptosis assay (**e**). All the experiments were performed biological triplicate. (*P < 0.05, **P < 0.01; independent t-test was used to compare control and treated cells)

Since high expression of piR-1245 indicates aggressive clinical behavior in CRC patients, we questioned whether piR-1245 plays a functional role in this malignancy. To this effect, we performed several functional assays to determine phenotypic alterations following overexpression or inhibition of piR-1245in colon cancer cells. We employed MTT assays to determine the proliferation rates of colon cancer cells transfected with piR-1245 mimics or antisense oligonucleotides. Our results revealed that inhibition of piR-1245 had a pronounced effect on suppressing growth proliferation of HCT116 and SW480 cells, while in contrast, overexpression of piR-1245 resulted in enhanced cell proliferation (Fig. 2a). Meanwhile, we also performed colony formation assays to evaluate the effect of piR-1245 on the colony-forming ability of single cells. As shown in Fig. 2b, inhibition of piR-1245 in HCT116 and SW480 cells demonstrated significantly reduced number of colonies compared to control cells, while up-regulation of piR-1245 markedly increased the total number of colonies. In line with above findings, inhibition of piR-1245 significantly reduces the percent of Ki-67 positive colon cancer cells, suggesting that piR-1245 functions as a positive regulator of cell survival (Fig. 2c and Additional file 6: Figure S4).

Since high expression of piR-1245 associated with lymph node and distant metastasis in CRC patients, we next interrogated whether it may regulate cell migration and invasion in colorectal cancer cells. As illustrated in Fig. 2d, inhibition of piR-1245 significantly suppressed cell migration and invasion in both HCT116 and SW480 cells compared to the control cells, confirming our findings in the clinical patient cohorts.

Resistance to programmed cell death is recognized as one of the cancer hallmarks that contributes to disease progression and eventual tumor metastasis [37]. Based on our clinical data, we hypothesized that piR-1245 also plays a key role in resistance to apoptosis in colorectal cancer cells. In line with our hypothesis, inhibition of piR-1245 significantly induced apoptosis in HCT116 and SW480 cells (Fig. 2e). Collectively, our data showed newly discovered piR-1245 exerts oncogenic function in CRC through promotion of cell survival, migration and invasion as well as suppression of apoptosis.

### The piR-1245 affects multiple cancer-related pathways involved in cell proliferation, cell death and apoptosis

**Fig. 3:**
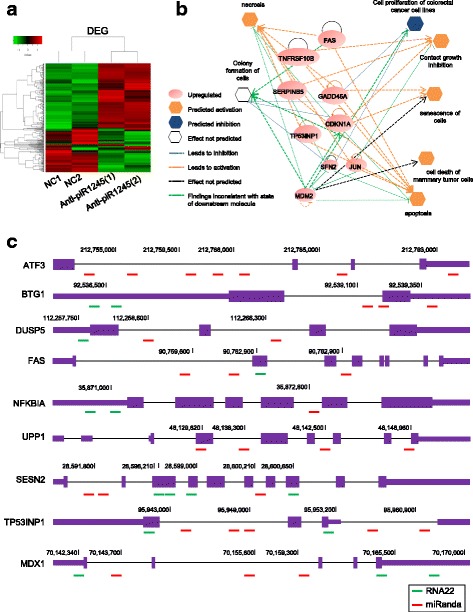
Identification of piR-1245 downstream target genes. **a** HCT116 cells were treated with or without piR-1245 antisense and subsequently performed gene expression microarray analysis. A total of 244 mRNAs were detected to be differentially expressed with a fold change of ≥ 1.5 and a corresponding *P* < 0.01. Notably, 168 genes were found to be up-regulated, while 76 genes were down-regulated in piR-1245-inhibited cells compared to control cells. **b** IPA analysis was performed for the upregulated genes to interrogate the function of piR-1245 in CRC. The results confirmed the putative model that activated p53 pathway, which was induced by piR-1245 inhibition, led to cell apoptosis, necrosis, cell death, contact growth inhibition, senescence of cells, and inhibited cell proliferation, colony formation. Furthermore, IPA showed the piR-1245 acts as important regulator of cell death and survival (**c**) miRANDA and RNA22 tool was used to predict the binding of piR-1245 to potential targets

To investigate the oncogenic mechanism of piR-1245 in CRC, we investigated its impact on transcriptomic alterations in CRC cell lines. We treated HCT116 cells with or without piR-1245 antisense and subsequently performed gene expression microarray analysis. As shown in Fig. 3a, a total of 244 mRNAs were detected to be differentially expressed with a fold change of ≥ 1.5 and a corresponding *P* < 0.01. Notably, 168 genes were found to be upregulated, while 76 genes were downregulated in piR-1245-inhibited cells compared to control cells.

Strikingly, the top 10 GO term enrichment analysis for upregulated genes favored cell death or apoptosis, cell proliferation, protein metabolic process and protein, while the downregulated genes were enriched for chromatin assembly and catalytic activity (Additional file 7: Figure S5).

In order to obtain further insights into disease and functional networks, we performed Ingenuity Pathway Analysis (IPA) based on our gene expression profiling results. The results confirmed the putative model that activated p53 pathway, which was induced by piR-1245 inhibition, led to cell apoptosis, necrosis, cell death, contact growth inhibition, senescence of cells, and inhibited cell proliferation, colony formation. Furthermore, IPA showed the piR-1245 acts as important regulator of cell death and survival (Fig. 3b). Based on these findings, these biological processes and molecular functions could contribute to the development of CRC.

### Identification of piR-1245 target genes in CRC

**Fig. 4:**
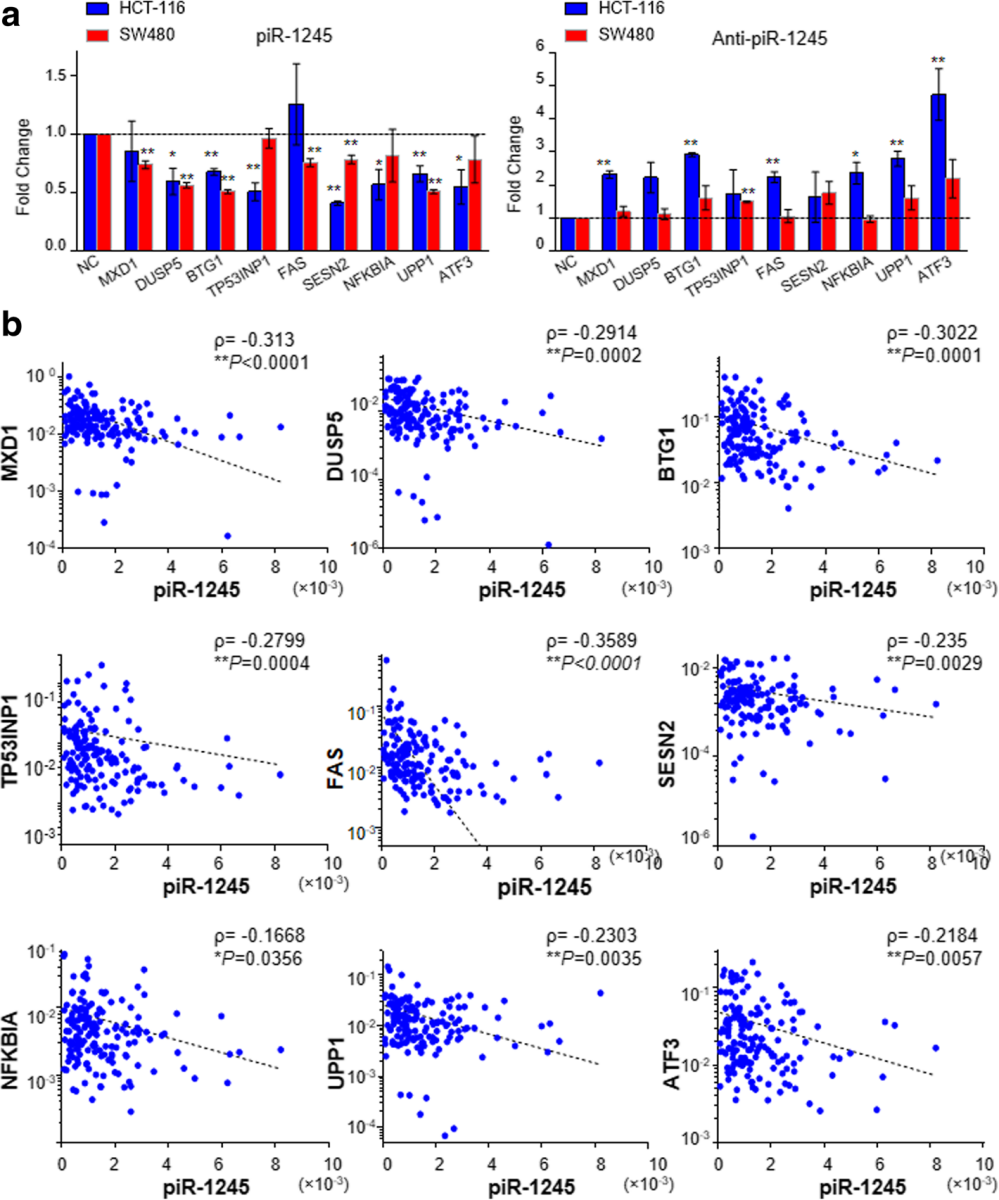
The correlation between piR-1245 and its target genes in CRC tissues. **a** qPCR was performed to confirm the expression change of target genes after piR-1245 overexpression or knockdown in HCT116 and SW480 cells. (*n* = 3, *P < 0.05, **P < 0.01, independent t-test was used to compare control and treated cells). **b** qPCR was performed to evaluate the expression correlation between piR-1245 and its targets in CRC tissues. (*n* = 159, *P < 0.05, **P < 0.01; Spearman’s rank correlation (ρ) was used for the correlation analysis)

A growing body of studies have shown that piRNAs have the capability to bind a diverse spectrum of downstream target genes by forming specific piRNA silencing complexes (pi-RISC), leading to RNA repression via imperfect base-pairing between the two types of RNAs [38–40]. We therefore examined potential target sites of piR-1245 interaction and the impacted downstream genes. We used miRANDA and RNA22 tool, applied stringent thermodynamic parameters and binding energy thresholds, to predict biologically relevant piRNA:mRNA interactions. We found there were at least 9 potential targets complementary to piR-1245including ATF3, BTG1, DUSP1, FAS, NFKBIA, UPP1, SESN2, TP53INP1 and MDX1. The examples of piRNA:mRNA complementarities identified by this approach are shown in Fig. 3c and Additional file 8: Figure S6 and Additional file 9: Figure S7.These genes have been reported to be involved in key cellular processes in CRC, including cell death and survival, cell cycle, DNA replication and repair or cell-cell communication (Additional file 10: Table S2). We further performed qPCR to confirm the expression change of target genes following piR-1245 overexpression or knockdown in HCT116 and SW480 cells and we are able to successively validate these findings (Fig. 4a). To further validate our in vitro results that piR-1245 regulated those tumor suppressors, we investigated the expression correlation between piR-1245 and its target genes in colorectal cancer tissues. Our results indicated that the expression of these targets were all negatively associated with piR-1245 expression in CRC (*P* < 0.05; Fig. 4b). Moreover, several genes demonstrated a strong inverse correlation with piR-1245 including MXD1, BTG1 and FAS, suggesting their expression levels are tightly synchronized with the piR-1245 function, and highlighting that piR-1245 serves as a potential key oncogenic regulator in CRC.

## Discussion

Colorectal cancer is one of the most common cancers worldwide. Consequently, elucidation of the molecular mechanisms underlying its progression is critical for the development of new diagnostic and prognostic biomarkers, as well as identification of better therapeutic targets for the management of patients with this deadly malignancy. Herein, we for the first time performed systematic piRNA expression profiling, and identified piR-1245, as a novel oncogenic piRNA mediating CRC pathogenesis. We have made several novel observations in this study. First, we discovered that piR-1245 is frequently overexpressed in CRC tissues from different cohorts, and its overexpression associated with several known risk clinicopathological factors including tumor differentiation and metastasis. Second, our data revealed that patients with high expression of piR-1245 had shorter overall survival, highlighting its applicability as a promising prognostic biomarker in CRC. Third, ours is the first study to demonstrate the biological relevance of this piR-1245 as a tumor-promoting noncoding RNA in CRC. Fourth, microarray analysis revealed thatpiR-1245 regulates several key cancer pathways, supporting its oncogenic role in CRC. Finally, we discovered several important tumor suppressors as direct downstream gene targets, and their expression was inversely correlated with the piR-1245, suggesting this small noncoding RNA promotes CRC development through inhibition of these target genes at the transcriptional level.

In contrast to the growing body of studies that underpin the miRNA-cancer connection, knowledge of piRNAs in tumorigenesis, particularly in CRC, remains currently in its infancy. PiRNAs are roughly 26-30 nucleotides in length and associate specifically with Argonaute proteins that belong to the PIWI subfamily [40, 41]. Although previously considered to be germline specific and guardians for protecting the integrity of the genome against transposon-induced insertional mutations [42], mounting evidence now point towards novel active role of piRNAs in somatic gene regulation, through other mechanisms such as transcriptional gene silencing and sequence-specific DNA methylation [16, 40, 41]. Interestingly, recent studies have demonstrated that piRNAs are widely expressed and play important roles in somatic cells [17]. Furthermore, few studies have begun to investigate the differentially expressed piRNAs in human cancers and their benign counterparts. It is noteworthy that piR-651 and piR-823 were found to be dysregulated in gastric cancer [43, 44], and moreover, recent study showed a panel of piRNAs are associated with prognosis in breast cancer [28], suggesting that piRNAs which previously considered as “junk” RNAs, are indeed involved in tumor progression and could be used as clinically-relevant biomarkers.

Until now, there are limited studies reporting the functional or clinical significance of piRNAs in CRC. We noted Cheng, et al. revealed piR-651 was overexpressed in several types of cancers including CRC [44]. However, the clinical and biologic significance of this piRNA in CRC remains unknown. In this study, through small RNA-seq analysis, we identified another specific piRNA, the piR-1245, which was consistently overexpressed in colorectal cancer tissues across different cohorts, highlighting its important role in CRC development. Notwithstanding its overexpression in cancer, we discovered that piR-1245 is a promising cancer biomarker, since its overexpression correlated with known risk clinicopathological features such as tumor depth, tumor differentiation and metastasis. Furthermore, another major finding of our study was that piR-1245 was a robust prognostic biomarker for survival prediction in CRC patients. These findings may help provide a better understanding of the mechanisms of piRNA in cancer progression and metastasis in CRC, and suggest that this novel small RNA may be an important disease biomarker and a potential therapeutic target in this disease.

To fully appreciate the clinical significance of piR-1245 in CRC, its biological significance as a contributor to colorectal pathogenesis should also be considered. Our functional experiments provide convincing evidence to support for the associations of piR-1245 with an aggressive clinical phenotype, where piR-1245 promotes CRC cells survival, migration and invasion as well as suppression of apoptosis. Consistent with this paradigm, our gene expression profiling results revealed that piR-1245 affects cancer-related pathways and functions as an oncogenic regulator. Accordingly, our results successfully proved our hypothesis, whereby overexpression of piR-1245 affected gene regulatory network for CRC and resulted in an aggressive phenotype, both biologically and clinically.

To further decipher the mechanic role of piR-1245 in CRC, we interrogated its potential downstream gene targets. By using bioinformatics approach, we identified nine ‘functionally relevant’ cancer-related genes. Interestingly, these nine candidates are involved in key tumor suppressive pathways and their expression inversely correlated with piR-1245 expression, supporting the oncogenic role of piR-1245 in CRC. Surprisingly, piR-1245 was found to not only bind to the exonic regions but also within the intronic regions. A recent study reported that piRNAs are able to bind to pre-mRNA introns and subsequently lead to the decay of targeted pre-mRNA through nuclear exosomes [38], suggesting that piR-1245 may use a similar mechanism to downregulate the expression of target genes. Furthermore, Watanabe, et al. suggested that piRNAs may suppress expression level of mRNAs harboring transposon sequence in 3’UTR or 5’UTR region [40]. Besides, piRNAs may also serve as natural antisense molecules that target genes by binding to their CDS regions or function as siRNAs to target 3’UTR [39–42]. In our study, we observed that piR-1245 could target 3’UTR, CDS or 5’UTR region via perfect or imperfect base-pairing between the two types of RNAs, by a mechanism that closely resembles that of antisense or siRNA. Although a number of possible scenarios could account for the interaction between piR-1245 and its target mRNAs, our data clearly demonstrated that the expression of these targets was significantly altered following gain or loss of piR-1245 expression in CRC cell lines.

## Conclusion

Our findings implicate piR-1245 as a potential modulator of colorectal carcinogenesis; a function possibly linked to piRNA-dependent mRNA degradation of its downstream targets. However, the precise mechanisms for the interaction between piR-1245 and its targets merit further investigation. To the best of our knowledge, these data represents first evidence for the role of piRNAs as prognostic biomarkers in CRC. Since piRNAs are abundant in cancer tissues, with improved profiling platforms and availability of tumor samples with extensive clinical annotations, it will be helpful to identify novel CRC-related piRNAs, which will further enhance our understanding of their mechanistic and prognostic contribution to this disease.

## Additional files

Additional file 1: Supplementary Methods. (DOCX 17 kb)

Additional file 2: Table S1. Primers sequence. (DOCX 13 kb)

Additional file 3: Figure S1. PIWIL1 and PIWIL4 are overexpressed in CRC A. Oncomine and Protein atlas database showed the mRNA and protein level of PIWIL1 and PIWIL4 are significantly overexpressed in CRC tissues compared to normal tissues. B. The representative IHC staining of PIWIL1 and PIWIL4 in CRC and normal tissues (provided by Protein atlas database). (TIFF 444 kb)

Additional file 4: Figure S2. The expression level of candidate piRNAs in validation cohorts. The expression of piR-1245 and piR-26,525 were further confirmed in the Shanghai cohort (cohort I) and subsequently validated in the Okayama cohort (cohort II). piR-1245 was consistently overexpressed in cancer vs. normal tissues in each cohort. **P < 0.01, Wilcoxon paired test. (TIFF 100 kb)

Additional file 5: Figure S3. The expression level of piR-1245 in different cancer type. The TCGA datasets showed piR-1245 is significantly up-regulated in tumor tissues compared to normal tissues in different types of cancer. **P < 0.01, Wilcoxon paired test. (TIFF 118 kb)

Additional file 6: Figure S4. The expression level of Ki-67 in CRC cell lines treated with piR-1245 overexpression or inhibition. HCT116 and SW480 cells were transfected with piR-1245 antisense, RNA oligos or control oligos. The representative images showed expression of Ki67 was detected by immunofluorescence assay from different treatment groups. The overexpression of piR-1245 in HCT116 cells showed increased level of proliferation marker Ki-67, while inhibition of piR-1245 showed decreased level of Ki-67, compared to the control cells. (TIFF 193 kb)

Additional file 7: Figure S5. Gene Ontology (GO) and disease functions of IPA analysis for the differential expressed genes in HCT116 treated with or without piR-1245 antisense treatment. Go annotation of top 10 enrichment pathways covering domains of biological processes, cellular components and molecular functions. The top 10 GO term enrichment analysis for up-regulated genes favored cell death or apoptosis, cell proliferation, protein metabolic process and protein, while the down-regulated genes were enriched for chromatin assembly and catalytic activity. IPA for the disease functions of piR-1245 in CRC showed that it correlates with cell death and survival. (TIFF 560 kb)

Additional file 8: Figure S6. Prediction of piR-1245’s target by miRanda. The representative images showed the binding sites between piR-1245 and its targets. (TIFF 197 kb)

Additional file 9: Figure S7. Prediction of piR-1245’s target by RNA22. The representative images showed the binding sites between piR-1245 and its targets. (TIFF 127 kb)

Additional file 10: Table S2. The function of piR-1245 targets and their expression in CRC. (DOCX 17 kb)
