# Supplementary material for: Novel evidence for a PIWI-interacting RNA (piRNA) as an oncogenic mediator of disease progression, and a potential prognostic biomarker in colorectal cancer
Source: Mol Cancer. 2018 Jan 30;17:16. doi: 10.1186/s12943-018-0767-3 (PMC5791351; doi:10.1186/s12943-018-0767-3)
Supplement: Supplementary file 1 — Supplementary Methods. (DOCX 17 kb) [file 12943_2018_767_MOESM1_ESM.docx]

**Identification of piRNA-1245 - a novel oncogenic piRNA associated with disease progression, and a promising prognostic biomarker in colorectal cancer**

Wenhao Weng^1,2,3#^, Na Liu^4#^, Yuji Toiyama^5^, Masato Kusunoki^5^, Takeshi Nagasaka^6^, Toshiyoshi Fujiwara^6^, Qing Wei^7^, Huanlong Qin^8^, Haifan Lin^4^ , Yanlei Ma^9,10^and Ajay Goel^1^

Supplementary Methods

**METHODS:**

Small RNA-sequencing analysis

For RNA-sequencing, 1μg of total RNA was used for library preparation with Illumina’s TruSeq small RNA sample preparation Kit. Linker sequences were trimmed off from the 50 nt raw sequences by using fastx_clipper with at least 8bp match. All the trimmed sequences were shorter than 9 nucleotides length. Next, the trimmed sequences were filtered for human piRNAs reported in the latest version of pirBase. The remaining small RNAs that mapped to human genome hg38 version were matched to known piRNAs collected from the piRNA bank (<http://pirnabank.ibab.ac.in>), and pirbase (<http://www.regulatoryrna.org/database/piRNA)>.DESeq was employed to identify differentially expressed piRNAs in colorectal cancer patients (with ≥2 fold change and *P*-value≤0.01).

piRNA quantification by qRT-PCR

Expression of identified piRNAs (DQ593356/piR-hsa-23619, DQ596309/piR-hsa-26525, DQ593752/piR-hsa-24000 and DQ570994/piR-hsa-1245) was analyzed using Custom TaqMan small RNA assays (Applied Biosystems, Foster City, CA, USA), and U6 expression was used as an endogenous control for data normalization, as described previously. The average expression levels of tissue piRNAs was normalized against U6 using the 2^-ΔCt^ method.

Gene expression analysis by quantitative Reverse Transcription Polymerase Reaction (qRT-PCR)

The qRT-PCR assays were performed using QuantStudio-6 Flex Real-Time PCR System (Applied Biosystems Foster City, CA). Five hundred ng of total RNA was converted to cDNA using High-Capacity cDNA Reverse Transcription Kit (Applied Biosystems, Foster City, CA). Real-time PCR was thereafter performed using Fast SYBR Green Master Mix (Applied Biosystems, Foster City, CA). The relative expression of target genes was determined by 2^-Δct^ method. GAPDH were used as normalizers. The primer sequences used are shown in **Supplementary Table S1**.

Cell lines, RNA oligos, antisense and transfection

HCT116 and SW480 were obtained from the American Type Culture Collection (ATCC, Rockville, MD) and cultured in Iscove’s modified Dulbecco’s medium (Invitrogen, Carlsbad, CA) supplemented with 10% fetal bovine serum (FBS) and antibiotics (100 U/ml penicillin and 100μg/ml streptomycin) at 37°C in 5% humidified CO2 atmosphere. These cell lines were periodically authenticated using a panel of genetic and epigenetic biomarkers.

For the overexpression of piR-1245 in CRC cell lines, HCT116 and SW480 were transfected in triplicates with either single-stranded RNA oligos (5’AGCCCUGAUGAUGCCCACUCCUGAGC-3’ with 2'-O-methylated 3'-end) or a single-stranded scrambled RNA controls (5’-UCA CAA CCU CCU AGA AAG AGU AGA -3’ with 2'-O-methylated 3'-end). For the inhibition of piR-1245 in CRC cell lines, we designed antisense oligos as described previously. The 2’-O-Me-modified antisense sequence was 5’-CUUA GCT CAG GAG TGG GCA TCA TCA GGG CT ACCUU-3’, while negative scrambled control was 5’-CUUATC aGG ACT gCTACt GGT GcG GAC gCGACCUU-3’.

For transfections, CRC cells were transfected with RNA oligos or antisense at a final concentration of 100 nmol/L using LipofectamineRNAiMAX (Invitrogen) and Opti-MEM (Gibco) according to the manufacturer's instructions.

MTT and colony formation assay

MTT (3-(4,5-dimethylthiazol-2-yl)-2,5-diphenyltetrazolium bromide) assay (Sigma, St. Louis, MO, USA) was used to evaluate cell proliferation. Cells were seeded at 1×10^3^ cells per well in 96 well plates. MTT was performed at 0, 24, 48 and 72h time-points. Optical density was measured at 570 nm using Infinite 200 Pro multi-reader (Tecan Group Ltd, Morrisville, NC). For the colony formation assay, 500 cells were seeded in each well of 6 well plates and incubated for 10 days. The colonies were stained with crystal violet and counted.

Cell invasion, migration and apoptosis assay

Migration and invasion assays were performed in Boyden chambers (Corning, Corning, NY) using 8 μm-size pore membrane coated with matrigel (for invasion assays) or without matrigel (for migration assays). Transfected cells in serum-free medium were seeded onto each insert at a density of 2x10^5^ cells/insert, with culture medium containing 10% FBS in the bottom well. Following 24h incubation, non-invading cells were removed by scraping the top of the membrane. Invaded cells on the bottom of the membrane were then fixed and stained using the Diff-quick staining kit (Thermo Scientific, Rockford, IL). Stained cells were counted using a light microscope. For apoptosis assays, Muse Annexin V and dead cell kits (Millipore, Billerica, MA) were used according to the manufacturer’s instructions.

Gene expression microarray analysis

To investigate the regulatory role of piR-1245 on genome-wide target mRNAs, we treated HCT116 cells with or without piR-1245 antisense, and subsequently performed Affymetrix GeneChip Human gene 2.0 ST arrays. The microarray probe intensity values (CEL files) were background corrected and normalized by Robust Multiarray Average (RMA) method. Comparison analysis was performed by using the LIMMA Bioconductor package to assess the differentially expressed mRNAs. The genes with ≥1.5 fold change and *P*-value≤0.01 were selected as candidate targets. We performed GO analysis in DAVID for these differentially expressed genes to evaluate their enrichment for specific biological functions. To gain further insight into piR-1245-related disease and function networks, we performed QIAGEN’s Ingenuity Pathway Analysis (www.qiagen.com/ingenuity).
