# Supplementary material for: Novel evidence for a PIWI-interacting RNA (piRNA) as an oncogenic mediator of disease progression, and a potential prognostic biomarker in colorectal cancer
Source: Mol Cancer. 2018 Jan 30;17:16. doi: 10.1186/s12943-018-0767-3 (PMC5791351; doi:10.1186/s12943-018-0767-3)
Supplement: Supplementary file 2 — Primers sequence. (DOCX 13 kb) [file 12943_2018_767_MOESM2_ESM.docx]

Table S1: Primers sequence

| Gene | Forward(5’→3’) | Reverse(5’→3’) |
| --- | --- | --- |
| MXD1 | GCTGAACATGGTTATGCCTCC | AGCCCGTCTATTCTTCTCCATTTC |
| DUSP5 | TCCTGAGTGTTGCGTGGATG | GGGCCACCCTGGTCATAAG |
| BTG1 | GGAGCTGCTGGCAGAACATTA | GTGCTGCCTGTCCAATCAGA |
| TP53INP1 | CTCACGGGCACAGAAGTGGAA | ATCCACTGGGAAGGGCGAA |
| FAS | GTACGGAGTTGGGGAAGCTC | ACAGACGTAAGAACCAGAGGT |
| SENS2 | TCGCTCTCCTCCTTCGTGTT | TCAAAGCCCCCAGAGTTGTTC |
| NFKBIA | CCCCTACACCTTGCCTGTG | CACGTGTGGCCATTGTAGTTG |
| UPP1 | GAGTGGGCTTGGTGAGGTG | CAGGACCCGTCAGAGGAGAG |
| ATF3 | GCCGAAACAAGAAGAAGGAGA | TCGTTCTTGAGCTCCTCAATC |
| GAPDH | TGTAGTTGAGGTCAATGAAGGG | ACATCGCTCAGACACCATG |
