# Supplementary figures and images for: Novel evidence for a PIWI-interacting RNA (piRNA) as an oncogenic mediator of disease progression, and a potential prognostic biomarker in colorectal cancer
Source: Mol Cancer. 2018 Jan 30;17:16. doi: 10.1186/s12943-018-0767-3 (PMC5791351; doi:10.1186/s12943-018-0767-3)

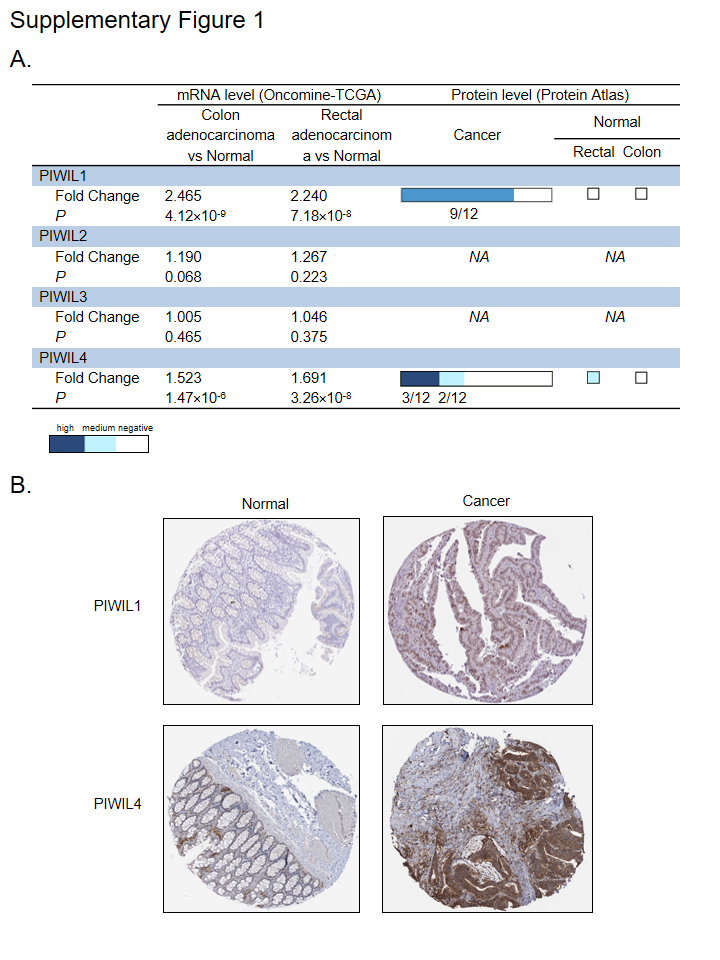

Supplement: Supplementary file 3 — PIWIL1 and PIWIL4 are overexpressed in CRC A. Oncomine and Protein atlas database showed the mRNA and protein level of PIWIL1 and PIWIL4 are significantly overexpressed in CRC tissues compared to normal tissues. B. The representative IHC staining of PIWIL1 and PIWIL4 in CRC and normal tissues (provided by Protein atlas database). (TIFF 444 kb) [file 12943_2018_767_MOESM3_ESM.tif]

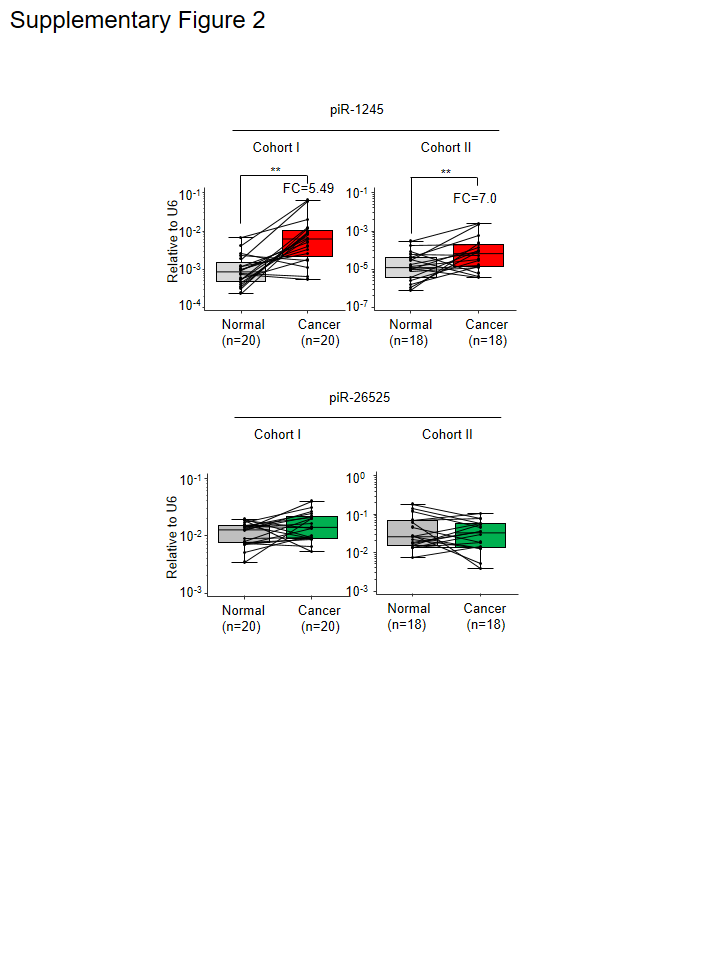

Supplement: Supplementary file 4 — The expression level of candidate piRNAs in validation cohorts. The expression of piR-1245 and piR-26,525 were further confirmed in the Shanghai cohort (cohort I) and subsequently validated in the Okayama cohort (cohort II). piR-1245 was consistently overexpressed in cancer vs. normal tissues in each cohort. **P < 0.01, Wilcoxon paired test. (TIFF 100 kb) [file 12943_2018_767_MOESM4_ESM.tif]

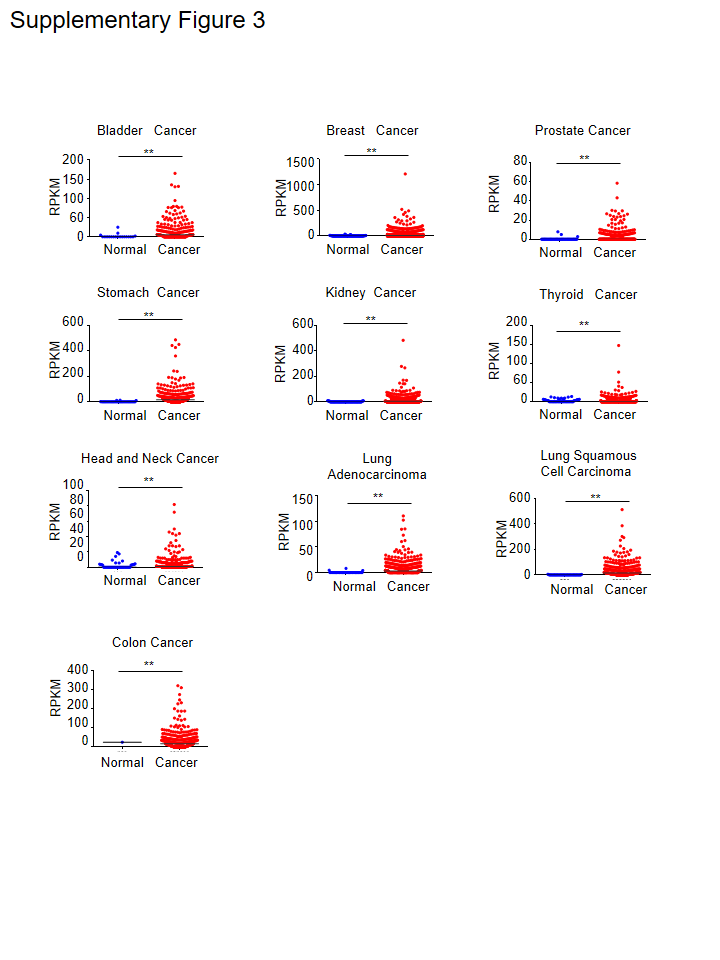

Supplement: Supplementary file 5 — The expression level of piR-1245 in different cancer type. The TCGA datasets showed piR-1245 is significantly up-regulated in tumor tissues compared to normal tissues in different types of cancer. **P < 0.01, Wilcoxon paired test. (TIFF 118 kb) [file 12943_2018_767_MOESM5_ESM.tif]

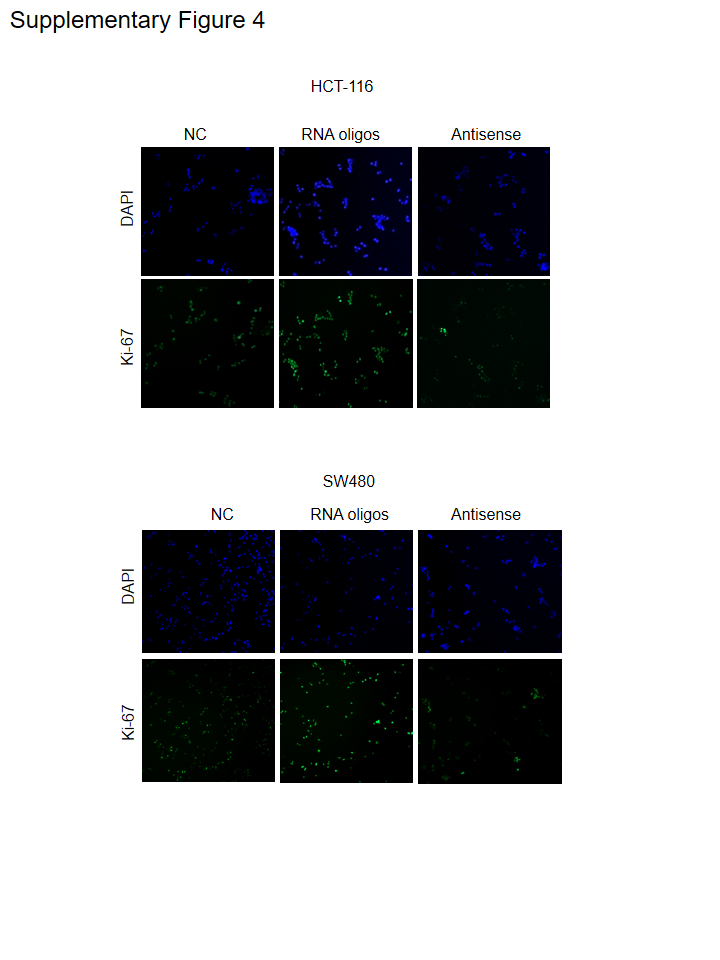

Supplement: Supplementary file 6 — The expression level of Ki-67 in CRC cell lines treated with piR-1245 overexpression or inhibition. HCT116 and SW480 cells were transfected with piR-1245 antisense, RNA oligos or control oligos. The representative images showed expression of Ki67 was detected by immunofluorescence assay from different treatment groups. The overexpression of piR-1245 in HCT116 cells showed increased level of proliferation marker Ki-67, while inhibition of piR-1245 showed decreased level of Ki-67, compared to the control cells. (TIFF 193 kb) [file 12943_2018_767_MOESM6_ESM.tif]

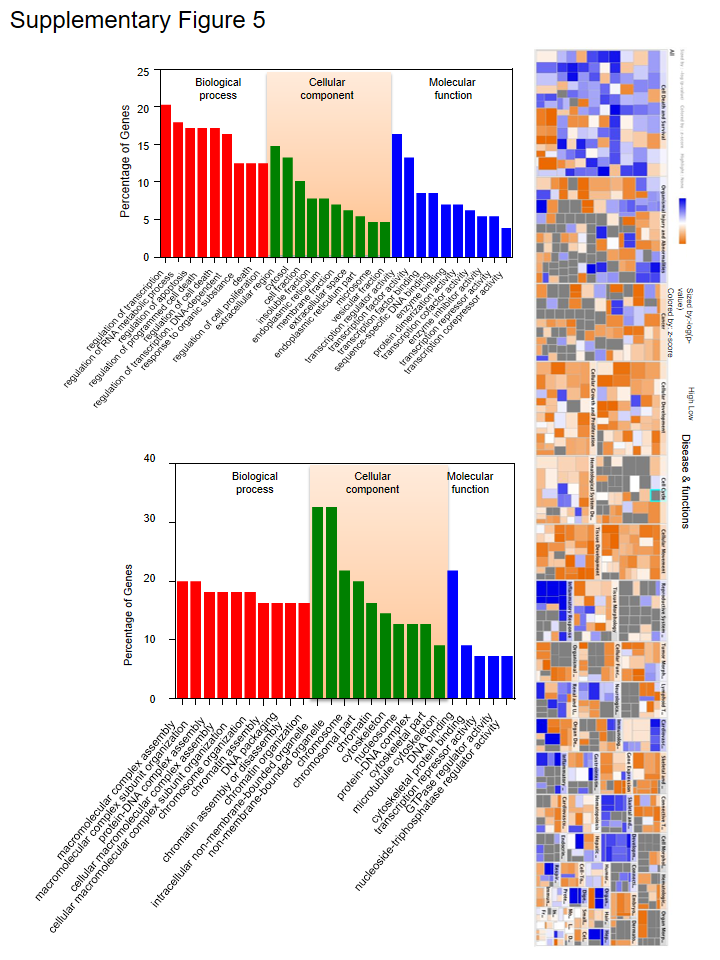

Supplement: Supplementary file 7 — Gene Ontology (GO) and disease functions of IPA analysis for the differential expressed genes in HCT116 treated with or without piR-1245 antisense treatment. Go annotation of top 10 enrichment pathways covering domains of biological processes, cellular components and molecular functions. The top 10 GO term enrichment analysis for up-regulated genes favored cell death or apoptosis, cell proliferation, protein metabolic process and protein, while the down-regulated genes were enriched for chromatin assembly and catalytic activity. IPA for the disease functions of piR-1245 in CRC showed that it correlates with cell death and survival. (TIFF 560 kb) [file 12943_2018_767_MOESM7_ESM.tif]

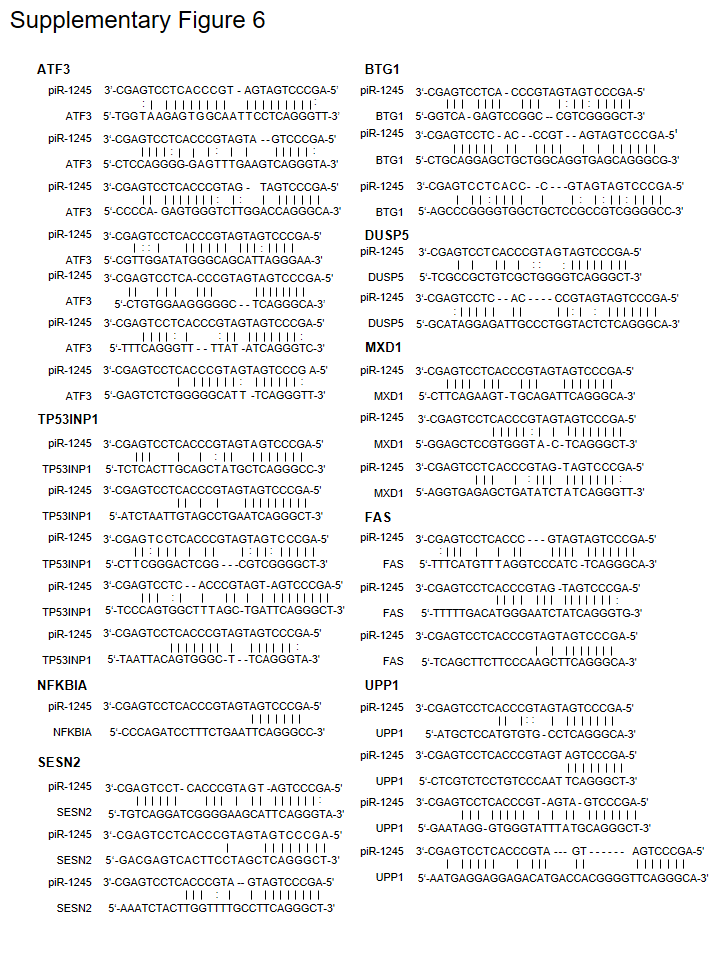

Supplement: Supplementary file 8 — Prediction of piR-1245’s target by miRanda. The representative images showed the binding sites between piR-1245 and its targets. (TIFF 197 kb) [file 12943_2018_767_MOESM8_ESM.tif]

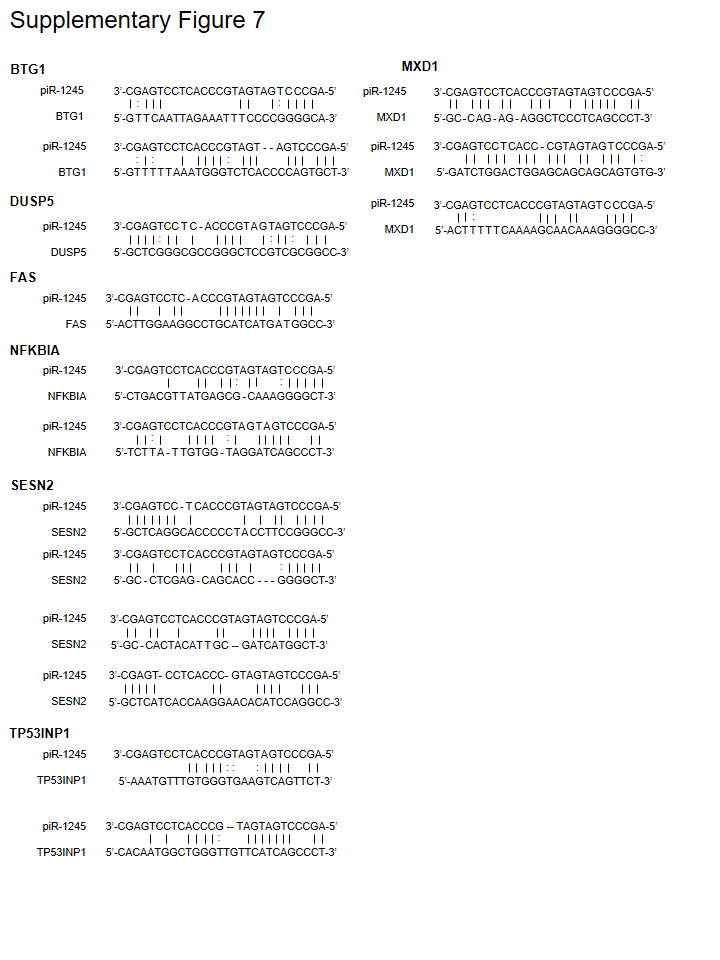

Supplement: Supplementary file 9 — Prediction of piR-1245’s target by RNA22. The representative images showed the binding sites between piR-1245 and its targets. (TIFF 127 kb) [file 12943_2018_767_MOESM9_ESM.tif]
