# Supplementary material for: Novel evidence for a PIWI-interacting RNA (piRNA) as an oncogenic mediator of disease progression, and a potential prognostic biomarker in colorectal cancer
Source: Mol Cancer. 2018 Jan 30;17:16. doi: 10.1186/s12943-018-0767-3 (PMC5791351; doi:10.1186/s12943-018-0767-3)
Supplement: Supplementary file 10 — The function of piR-1245 targets and their expression in CRC. (DOCX 17 kb) [file 12943_2018_767_MOESM10_ESM.docx]

Table S2: The function of piR-1245 targets and their expression in CRC

| Gene | Expression* | Function** | Process** | Component** |
| --- | --- | --- | --- | --- |
| MXD1 | down | RNA polymerase II core promoter proximal region sequence-specific DNA binding  protein binding  protein dimerization activity  transcription cofactor activity  transcription corepressor activity  transcription factor activity, sequence-specific DNA binding  transcriptional repressor activity, RNA polymerase II core promoter proximal region sequence-specific binding | cell proliferation  multicellular organism development  negative regulation of transcription from RNA polymerase II promoter  transcription, DNA-templated | nuclear chromatin  nucleus |
| DUSP5 | down | MAP kie tyrosine/serine/threonine phosphae activity  phosphae activity  protein binding  protein tyrosine phosphae activity  protein tyrosine/serine/threonine phosphae activity  protein tyrosine/serine/threonine phosphae activity | MAPK cascade  activation of MAPK activity  dephosphorylation  endoderm formation  inactivation of MAPK activity  peptidyl-threonine dephosphorylation  peptidyl-tyrosine dephosphorylation  protein dephosphorylation | nucleoplasm |
| BTG1 | down | enzyme binding  kie binding  protein binding  transcription cofactor activity | cell migration  negative regulation of cell growth  negative regulation of cell proliferation  positive regulation of angiogenesis  positive regulation of endothelial cell differentiation  positive regulation of fibroblast apoptotic process  positive regulation of myoblast differentiation  positive regulation of myoblast differentiation  regulation of transcription, DNA-templated | cytoplasm  cytoplasm  nucleus  nucleus |
| TP53INP1 | down | antioxnt activity  protein binding | apoptotic process  autophagic cell death  autophagosome assembly  cell cycle arrest  cellular oxnt detoxification  cellular response to  cellular response to ethanol  cellular response to hydroperoxide  cellular response to methyl methanesulfonate  negative regulation of cell migration  negative regulation of cell proliferation  positive regulation of apoptotic signaling pathway  positive regulation of autophagy  positive regulation of transcription, DNA-templated  regulation of apoptotic process  regulation of signal transduction by p53 class mediator  response to heat  response to stress  transcription, DNA-templated | PML body  autophagosome  cytoplasm  cytoplasmic vesicle  cytosol  nucleoplasm  nucleus |
| FAS | down | identical protein binding  kie binding  protease binding  protein binding  protein complex binding  receptor activity  signal transducer activity  tumor necrosis factor-activated receptor activity | B cell mediated immunity  activation of cysteine-type endopeptse activity involved in apoptotic process  activation of cysteine-type endopeptse activity involved in apoptotic signaling pathway  activation-induced cell death of T cells  aging  apoptotic process  apoptotic signaling pathway  brain development  cellular response to cobalt ion  cellular response to estrogen stimulus  cellular response to glucose stimulus  cellular response to hydrogen peroxide  cellular response to hydrostatic pressure  cellular response to hyperoxia  cellular response to hypoxia  cellular response to interleukin-1  cellular response to lithium ion  cellular response to mechanical stimulus  cellular response to phenylalanine  chordate embryonic development  circadian rhythm  dendrite regeneration  extrinsic apoptotic signaling pathway  extrinsic apoptotic signaling pathway in absence of ligand  extrinsic apoptotic signaling pathway via death domain receptors | CD95 death-inducing signaling complex  apical dendrite  apical plasma membrane  cell surface  cytoplasm  cytosol  death-inducing signaling complex  external side of plasma membrane  extracellular exosome  extracellular space  integral component of plasma membrane  membrane raft  neuron projection  neuronal cell body  nucleus  nucleus  perinuclear region of cytoplasm  plasma membrane  plasma membrane  plasma membrane |
| SESN2 | down | leucine binding  oxidoreduce activity, acting on peroxide as acceptor  protein binding  sulfiredoxin activity  NOT sulfiredoxin activity | DNA damage response, signal transduction by p53 class mediator  autophagy  cellular oxnt detoxification  NOT cellular oxnt detoxification  cellular oxnt detoxification  cellular response to amino acid stimulus  cellular response to leucine  cellular response to oxtive stress  fatty acid beta-oxtion  glucose ort  mitochondrial DNA metabolic process  negative regulation of TORC1 signaling  negative regulation of cell growth  negative regulation of translation in response to endoplasmic reticulum stress  positive regulation of macroautophagy  positive regulation of protein localization to nucleus  positive regulation of transcription from RNA polymerase II promoter in response to oxtive stress  protein kie B signaling  reactive oxygen species metabolic process  regulation of cAMP-dependent protein kie activity  regulation of gluconeogenesis involved in cellular glucose homeosis  regulation of protein phosphorylation  regulation of response to reactive oxygen species  response to glucose  response to insulin | colocalizes_with ATG1/ULK1 kie complex  colocalizes_with GATOR2 complex  colocalizes_with TORC2 complex  cytoplasm  cytosol  mitochondrion  colocalizes_with nucleotide-activated protein kie complex  nucleus |
| NFKBIA | down | NF-kappaB binding  NF-kappaB binding  enzyme binding  heat shock protein binding  identical protein binding  nuclear localization sequence binding  protein binding  protein complex binding  transcription factor binding  ubiquitin protein ligase binding | Fc-epsilon receptor signaling pathway  T cell receptor signaling pathway  apoptotic process  cellular response to cold  cellular response to cytokine stimulus  cellular response to organic cyclic compound  cytoplasmic sequestering of NF-kappaB  cytoplasmic sequestering of transcription factor  lipopolysaccharide-mediated signaling pathway  negative regulation of DNA binding  negative regulation of NF-kappaB transcription factor activity  negative regulation of Notch signaling pathway  negative regulation of apoptotic process  negative regulation of ld storage  negative regulation of macrophage derived foam cell differentiation  negative regulation of myeloid cell differentiation  nucleotide-binding oligomerization domain containing 1 signaling pathway  nucleotide-binding oligomerization domain containing 2 signaling pathway  positive regulation of NF-kappaB transcription factor activity  positive regulation of cellular protein metabolic process  positive regulation of cholesterol efflux  positive regulation of transcription from RNA polymerase II promoter  positive regulation of transcription from RNA polymerase II promoter  protein ort into nucleus, translocation  regulation of NF-kappaB ort into nucleus | I-kappaB/NF-kappaB complex  cytoplasm  cytosol  nucleus  plasma membrane |
| UPP1 | down | uridine phosphorylase activity | UMP salvage  cellular response to glucose starvation  nucleobase-containing compound metabolic process  nucleotide catabolic process  pyrimidine nucleoside catabolic process  pyrimidine nucleoside salvage  uridine catabolic process | cytosol |
| ATF3 | down | RNA polymerase II core promoter proximal region sequence-specific DNA binding  RNA polymerase II regulatory region sequence-specific DNA binding  identical protein binding  protein binding  protein heterodimerization activity  protein homodimerization activity  transcription corepressor activity  transcription factor activity, RNA polymerase II core promoter proximal region sequence-specific binding  transcription factor activity, sequence-specific DNA binding  transcription regulatory region DNA binding  transcription regulatory region sequence-specific DNA binding  transcriptional activator activity, RNA polymerase II transcription regulatory region sequence-specific binding  transcriptional repressor activity, RNA polymerase II core promoter proximal region sequence-specific binding | PERK-mediated unfolded protein response  cellular response to amino acid starvation  gluconeogenesis  negative regulation of ERK1 and ERK2 cascade  negative regulation of transcription from RNA polymerase II promoter  positive regulation of TRAIL-activated apoptotic signaling pathway  positive regulation of cell proliferation  positive regulation of transcription from RNA polymerase II promoter  positive regulation of transcription from RNA polymerase II promoter in response to endoplasmic reticulum stress  regulation of transcription from RNA polymerase II promoter in response to arsenic-containing substance  skeletal muscle cell differentiation  transcription from RNA polymerase II promoter | CHOP-ATF3 complex  nucleolus  nucleoplasm  nucleus |

*To compare the expression of target genes in cancer and normal tissues, Oncomine database was analyzed (Rhodes, D.R., et al. ONCOMINE: a cancer microarray database and integrated data-mining platform. Neoplasia,2004 6, 1-6).

** The function of target gene was provided by Gene Ontology Annotation (UniProt-GOA) Database
